# Genetic marker data for sweetpotato improvement

**DOI:** 10.1016/j.dib.2025.111630

**Published:** 2025-05-07

**Authors:** Imana L. Power, Brian E. Scheffler, Xiaofen F. Liu, Sheron A. Simpson, Linda L. Ballard, Marshall C. Lamb, Renee S. Arias

**Affiliations:** a Louisiana State University, AgCenter, Baton Rouge, LA 70803, USA; b USDA-ARS Genomics and Bioinformatics Research Unit, 141 Experiment Station Rd., Stoneville, MS 38776, USA; c USDA-ARS National Peanut Research Laboratory, 1011 Forrester Dr. S.E., Dawson, Georgia 39842, USA

**Keywords:** Microsatellites, SSR, SNP, *Ipomoea batatas*

## Abstract

A total of 768 molecular markers were developed for *Ipomoea batatas* (L.) Lam., consisting of 689 simple sequence repeats (SSRs) and 79 single nucleotide polymorphisms (SNPs). All the markers were distributed across the sweet potato genome, averaging 51 markers per chromosome. The markers were tested on DNA samples from five cultivars of *I. batatas*, assessing their amplification efficiency and polymorphism. Here we provide the primer sequences tested, their chromosome locations, the analysis including amplicon sizes, and highlight 92 that showed polymorphism between Beauregard and Tanzania. This dataset offers valuable resources for constructing high-resolution linkage maps and facilitates advanced genetic studies and breeding programs in sweetpotato.

Specifications Table

**Table:** 

Subject	Biological Sciences.
Specific subject area	Plant Science.
Type of data	Sequences in FASTA format, Primers list in Excel, Amplicons on five cultivars in excel, Figure Distribution of Markers per chromosome in Power Point, Figure Distribution of Chromosomes on chromosomes in Power Point.
Data collection	A total of 768 primer sets were generated using Primer3 [1] for simple sequence repeats (SSR) to generate amplicons between 100-200 bp, and tested on five *I. batatas* cultivars: Tanzania, Beauregard, 538300, 286619 and SC1149 using capillary electrophoresis as described before [2], run in an Applied Biosystems 3730 (Applied Biosystems) instrument, and the results manually extracted using GeneMapper 3.7 (Applied Biosystems). The chromosome location of the sequences that gave origin to the markers was searched by mapping the FASTA file to the *I. batatas* genome accession PRJNA301667 using CLC_Genomic Workbench V.24 (Qiagen). Both, primer sequences and their chromosome locations are provided.
Data source location	Sequence information and data were placed in a public repository: Harvard Dataverse.
Data accessibility	Repository name: Harvard Dataverse… Data identification number: https://doi.org/10.7910/DVN/YFJ0AK Direct URL to data: https://doi.org/10.7910/DVN/YFJ0AK
Related research article	The initial SSR enriched libraries were prepared [3]. The primer sequences, screening on *I. batatas* DNA and location on chromosomes have not been reported before. Optimized construction of microsatellite-enriched libraries: https://doi.org/10.1111/j.1755-0998.2009.02802.x

## 1 Value of the Data

• Generating SSR-enriched libraries, ordering primers, and screening markers has a high cost, therefore, the preliminary screening performed in the present work will make the use of markers for *I. batatas* more effective, for example, 275 of the 768 markers tested resulted in no amplification, and another 70 were of low quality. By reporting the results of allele calling, the reader can choose the effective markers to use.
• A current linkage map for *I. batatas* based on 210 EST-SSR markers has a resolution of 7.2 centimorgan (cM) [4]. Adding 490 new markers could help increase the linkage map resolution to 2.2 cM.
• Developing SSR markers is costly, time consuming and labour intensive (4), thus, the information reported here can help sweet potato breeders choose markers to use based on their quality, sequence homology to genes of interest, and chromosome location.
• A total of 79 markers reported here correspond to predicted SNPs; being *I. batatas* a hexaploid, SNPs related to SSRs can provide additional information regarding specific alleles.

## 2 Background

Sweetpotato, [*Ipomoea batatas* (L.) Lam.] is considered a “superfood” and is the 7^th^ most important food crop in the world [5]. Genetic improvement of the hexaploid (2n = 6x = 90) sweetpotato has been significantly constrained because its ploidy complicates traditional and molecular breeding efforts due to genome complexity, genetic redundancy, and difficulties in achieving stable inheritance of desirable traits [6]. Another challenge in sweetpotato improvement is the limited availability of genetic markers. The hexaploid genome of sweetpotato contains gene duplication and epistatic interactions that complicate the identification of genotype-phenotype relationships, as traits cannot easily be linked to single genes [7]. Simple Sequence Repeat (SSR) and Single Nucleotide Polymorphism (SNP) markers are recognized as powerful tools in breeding for traits of interest, and many of them have been identified in *I. batatas* as related to beta-carotene and starch content [8]. SSR markers consist of short, tandemly repeated DNA sequences with high polymorphism and co-dominant inheritance, while SNP markers are single base-pair variations in the genome, are abundant, and highly stable [9]. The objective of developing the SSR and SNP markers for sweetpotato facilitates a more saturated linkage map for sweetpotato, enabling more precise marker-assisted selection in breeding programs.

## 3 Data Description

**Fig. 1:**
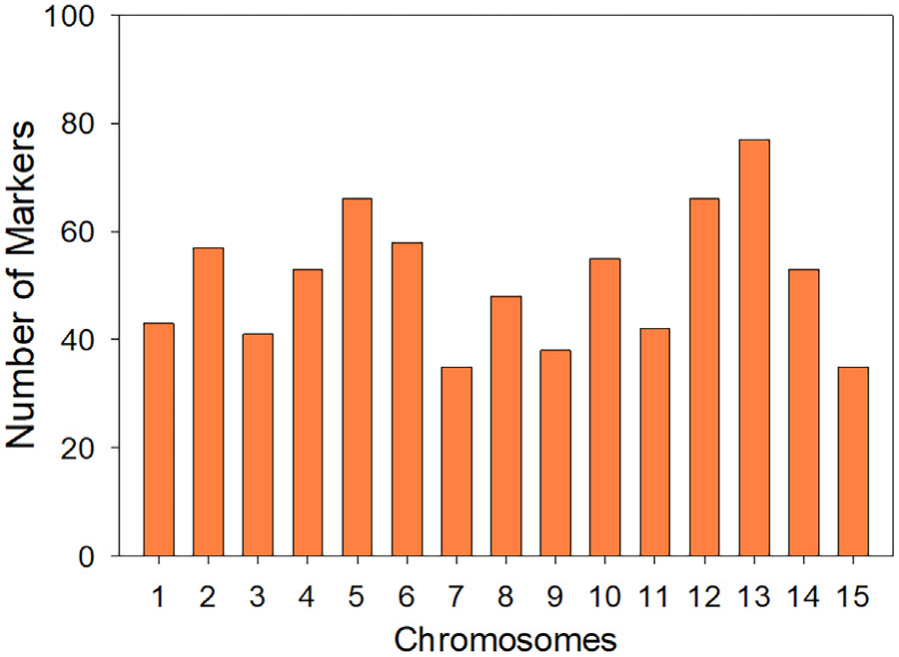
Distribution of Markers per *I. batatas* chromosome.

**Fig. 2:**
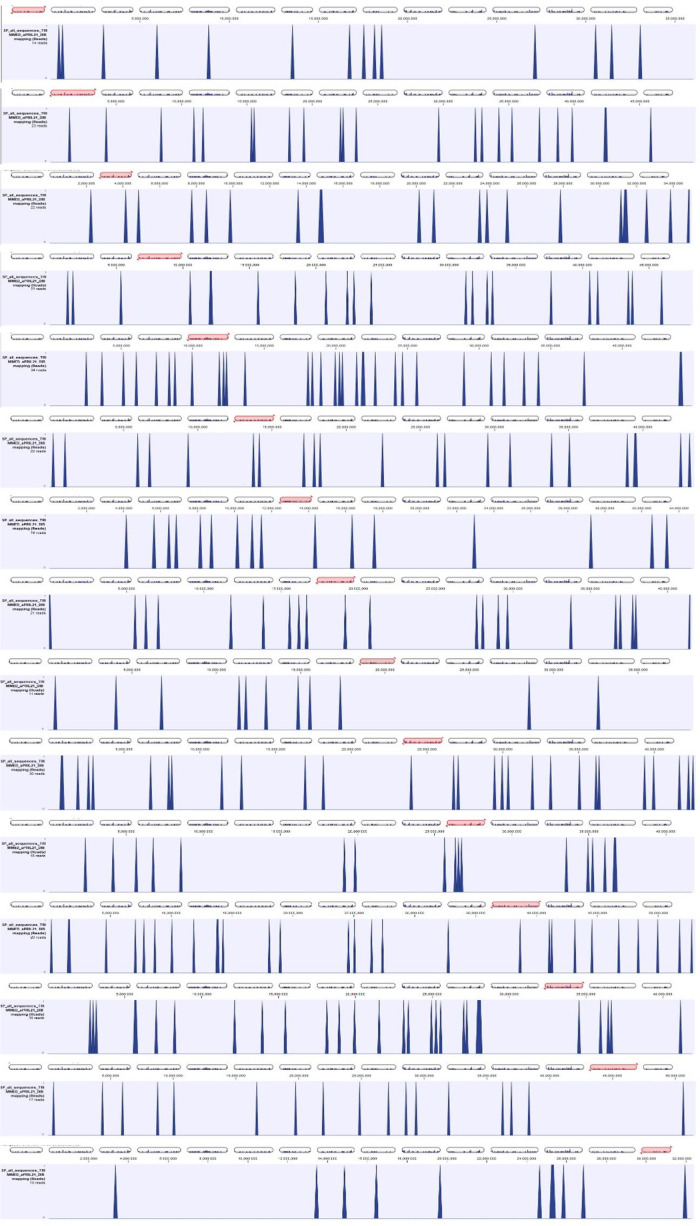
Distribution of Contigs on *I. batatas* chromosomes.

All data are available https://doi.org/10.7910/DVN/YFJ0AK. Sequences obtained from *Ipomoea batatas* SSR-enriched libraries are in SP_all_sequences_DIB.FASTA. Using these sequences, microsatellite mining was performed using SSR_Finder and Sputnik, followed by primer design on Primer3 [1] with the following parameters: optimum length 24 nt, optimum annealing temperature 62°C, maximum overlapping of SSR with primer sequence 5 nt, optimum amplicon size 120 nt. Those sequences that allowed designing primers for SSR detection were mentioned in Techen et al 2010 [3]. During the assemby of contigs containing SSRs multiple SNPs were observed, in these cases, more than one forward or reverse primer were designed to include the SNP at the 3′ end. The complete list of primers designed is provided as SPPLUS Primers to Order 8 plates_DIB.xlsx. Obtained markers were tested on five *I. batatas* genotypes: Tanzania, Beauregard, 538300, 286619 and SC1149 using capillary electrophoresis, run in an Applied Biosystems 3730 instrument, and the results manually extracted using GeneMapper 3.7; the detailed manual scoring of markers is shown on file SSR_SNP_Analysis_DIB.xlsx, a filtrable version of this table SSR_SNP_Analysis_Filtrable_DIB.xlsx is also provided. Marker sequences were mapped to the *I. batatas genome* (BioProject PRJNA301667) using CLC_Genomic Workbench V.24, and their distribution per chromosome is displayed in Fig. 1_Distribution_Markers_DIB.pptx and Fig. 2_Distribution_of_Contigs_on Chromosomes_DIB.pptx.

## 4 Experimental Design, Materials and Methods

Sweetpotato variety 'Beauregard' (B-24) was used to generate simple sequence repeat (SSR)-enriched libraries, using 25 biotinylated oligonucleotide repeats. In total 4,608 clones were sequenced of which 2,593 were assembled into contigs, identifying 2,551 repeats. This process led to the design of 768 markers, including 79 allele-specific single nucleotide polymorphisms (SNPs), some of which exhibited polymorphism between the cultivars 'Tanzania' and 'Beauregard'.

Out of the 768 markers, 275 failed to amplify, and 70 produced ambiguous results, leaving 423 effective markers. When combined with the 210 markers previously used to map the 1,508 centimorgan (cM) Ipomoea genome, the marker density could be increased from one marker per 7.2 cM to one per 2.4 cM. Notably, 325 markers successfully amplified across all five tested sweetpotato DNA samples.

BLAST analysis of the contigs against the *I. batatas* chromosomes revealed an average distribution of 167 ± 32 contigs per chromosome, ranging from 116 to 218 contigs per chromosome. This equates to approximately 25 to 55 markers per chromosome, with an average of 51.

In the primer design, a 3′ GC clamp was incorporated to enhance specificity. In addition, SSR_Finder and Sputnik were used to mine the sequences for microsatellite. Testing these markers across five sweetpotato DNA samples, resulted in 92 markers that could distinguish between the 'Beauregard' and 'Tanzania' cultivars, with 79 being allele-specific.

## Limitations

Not applicable*.*

## Ethics Statement

All authors have read and followed the ethical requirements for publication in Data in Brief and confirm that the current work does not involve human subjects, animal experiments, or any data collected from social media platforms.

## CRediT Author Statement

**Renee S. Arias**: Conceptualization, Methodology, Formal analysis, Visualization, Writing-original draft, review and editing; **Marshall C. Lamb**: Funding acquisition, Supervision; **Brian E. Scheffler**: Methodology, Data curation, Formal Analysis; **Xiaofen F. Liu**: Methodology, Data curation, Formal Analysis; **Sheron A. Simpson**: Methodology, Data curation, Formal Analysis; **Linda L. Ballard**: Methodology, Data curation, Formal Analysis; **Imana L. Power**: Writing-original draft, review and editing.

## Data Availability

DataverseSweetpotato SSR and SNP markers (Original data)
